# The pupil response is sensitive to divided attention during speech processing

**DOI:** 10.1016/j.heares.2014.03.010

**Published:** 2014-04-05

**Authors:** Thomas Koelewijn, Barbara G. Shinn-Cunningham, Adriana A. Zekveld, Sophia E. Kramer

**Affiliations:** a Section Audiology, Dept. of Otolaryngology-Head and Neck Surgery and EMGO Institute for Health and Care Research, VU University medical center, Amsterdam, The Netherlands; b Department of Biomedical Engineering, Center for Computational Neuroscience and Neural Technology, Boston University, Boston, USA; c Linnaeus Centre HEAD, Department of Behavioral Sciences and Learning, Linköping University, Linköping, Sweden

## Abstract

Dividing attention over two streams of speech strongly decreases performance compared to focusing on only one. How divided attention affects cognitive processing load as indexed with pupillometry during speech recognition has so far not been investigated. In 12 young adults the pupil response was recorded while they focused on either one or both of two sentences that were presented dichotically and masked by fluctuating noise across a range of signal-to-noise ratios. In line with previous studies, the performance decreases when processing two target sentences instead of one. Additionally, dividing attention to process two sentences caused larger pupil dilation and later peak pupil latency than processing only one. This suggests an effect of attention on cognitive processing load (pupil dilation) during speech processing in noise.

## 1. Introduction

Talking to friends in a bar can be an effortful task. There is music, there are often conversations going on in the background, and when some friends are in a heated discussion they might all start talking at the same time. During these complex listening conditions we must detect and select relevant target speech while inhibiting background sounds. Furthermore, we need to divide our attention based on how many persons of interest are talking. Unfortunately, listening to speech in adverse conditions often results in high cognitive load (Desjardins and Doherty, 2013; Gosselin and Gagné, 2011; Zekveld et al., 2011), which can cause listeners to lose track of a conversation. Mattys et al. (2012) define cognitive load as “any factor placing unusually high demands on central attentional and mnemonic capacities”. Attentional capacity is used for inhibiting distracting information; in contrast, working memory resources are recruited when pieces of information need to be combined or otherwise manipulated. Mattys et al. (2012) distinguish cognitive load arising from segregating speech from noise and cognitive load arising from extrinsic demands, such as dual tasking. Research confirms that different types of background sounds not only affect speech perception (Carhart et al., 1969; Festen and Plomp, 1990) but also cognitive load (Desjardins and Doherty, 2013; Koelewijn et al., 2012a). Additionally, recent studies show that working memory capacity is related to speech perception (Desjardins and Doherty, 2013; Ng et al., 2013; e.g., Rudner et al., 2011) and cognitive load (Desjardins and Doherty, 2013; Koelewijn et al., 2012b; e.g., Picou and Ricketts, 2011). Despite these studies showing the effect perceptual processes and working memory capacity have on load during speech perception, it is not yet known how divided attention affects cognitive processing load when listening to speech in noise.

Attention is the main cognitive process that allows us to focus on relevant speech (Best et al., 2006; Best et al., 2010; Cherry, 1953). Features like voice characteristics and semantic content help us to disentangle different streams of information (Bronkhorst, 2000). By directing attention we are able to focus on a speaker and on the audible parts of speech (Ruggles et al., 2011), which improves speech intelligibility (Allen et al., 2009; Ihlefeld and Shinn-Cunningham, 2008). Dividing attention over multiple streams of incoming speech affects performance as well. In a study by Best et al. (2010), a dichotic listening task was used to study the effect of divided attention on speech processing. A different sentence was presented to each ear simultaneously, with each sentence masked by an independent speech-shaped noise. One of the sentences was spoken by a female voice, the other by a male voice. There was a “single-task” condition in which participants were asked to report two target words embedded in a sentence spoken by the female voice and ignore two target words embedded in a sentence spoken by the male voice. Additionally, there was a “dual-task” condition in which participants were instructed to first report the target words by the female voice (S1), and then to report the target words spoken by the male voice (S2). Note that in both conditions the stimuli were constructed identically; the only difference between conditions was the task instruction. The results showed that performance on S1 was lower in the dual-sentence condition compared to the single-sentence condition; performance on S2 was even lower than performance on S1. These results suggest that dividing attention between concurrent stimuli comes with a strong cost in performance.

The observed differences in S1 performance between the single-sentence and dual-sentence condition could be explained by the “load theory” suggested by Lavie et al. (2004). According to this theory attentional mechanisms operate at two stages. First there is a filter at an early passive perceptual stage where high load, as a consequence of target processing, prevents distractors from being processed, which in turn leads to better performance. Secondly, there is an active cognitive stage where high load, as a result of tasks demand, affects late selection processes, which leads to a drop in performance. Note that this theory predicts that performance depends on both perceptual and cognitive load; cognitive load is of specific interest in the current study. In the study by Lavie et al. (2004), working memory load was manipulated while participants performed a selective attention task. This resulted in fewer resources available for late selection processes, which resulted in a drop in performance. In line with the load theory, the results of Best et al. (2010) show a drop in performance for the dual-task compared to the single-task condition. However, in this study the dual-task condition involved a divided attention task in contrast to an additional working memory task. In the divided attention condition it was the late selection process itself that was manipulated, which might have resulted in increased cognitive load. Although Best et al. (2010) observed a drop in performance, interactions between attention and cognitive load might be complex. While the load theory states that cognitive load affects selective attention, dividing attention could in turn have an effect on cognitive load. Best et al. (2010) did not specifically manipulate perceptual load, although speech-in-noise was presented at different SNRs. In contrast to the idea that increased perceptual load should lead to better performance (Lavie et al., 2004), a drop in SNR generally shows a drop in performance (e.g., Festen and Plomp, 1990), an effect also observed in Best et al. (2010). This can be explained by the fact that in the auditory domain, additional perceptual load in the form of irrelevant sound will often lead to masking, which is not necessarily the case for visual processing. Note that SNR as manipulated in Best et al. (2010) does not purely affect perceptual load as it also leads to changes in working memory load (Lunner and Sundewall-Thorén, 2007; Rönnberg et al., 2013).

The idea that masking affects cognitive load is in line with pupillometry research that shows that the pupil dilation response is affected by the level of intelligibility and the type of masker when processing speech in noise (Koelewijn et al., 2012a; Kramer et al., 1997; Zekveld et al., 2010). Pupil size increases when cognitive load increases (Beatty, 1982; Kahneman and Beatty, 1966), and is associated with intelligence measures that are supposed to reflect cognitive effciency (Ahern and Beatty, 1979). Additionally, the pupil dilation response is related to speech processing at a more cognitive level. For instance, the peak pupil dilation is larger when processing speech masked by an interfering talker compared to fluctuating noise (Koelewijn et al., 2012a). Note that the way pupil size responds to adverse listening conditions is consistent with the concept of cognitive load as defined by Mattys et al. (2012). This result was partly explained by inter-individual differences in working memory capacity and partly by inter-individual differences in the ability to inhibit irrelevant information or linguistic closure (Koelewijn et al., 2012b; Zekveld et al., 2011). Nonetheless, the amount of variance accounted for by these cognitive measures, in regression models explaining the peak pupil dilation during listening, was small (Koelewijn et al., 2012a). Hence, it is still not entirely clear what other cognitive processes are responsible for pupil dilation during speech processing in adverse listening conditions, and whether attention is one of these processes.

However, effects of inhibitory processes on the pupil response (Koelewijn et al., 2012a) suggest that attention-related processes contribute to cognitive load during processing of speech in noise. Additionally, the deployment of attentional processes has been shown to result in pupil dilation in humans and other primates (Hoeks and Levelt, 1993; Iriki et al., 1996). In all, previous research indicates that the use of attentional resources could add cognitive load during processing of speech in noise.

In the current study we investigated whether dividing attention over two streams of information leads to additional cognitive load compared to selectively focusing attention on only one stream and ignoring the other (a design based on that used in Best et al., 2010). This was tested at different levels of masking. We hypothesized that a lower SNR would result in a drop in performance as a result of increased masking, and that it would affect the overall cognitive load by increasing working memory load. To measure cognitive load, the participant’s pupil dilation response was recorded during the performance of each trial. Both mean and peak pupil dilation were used as physiological markers of cognitive processing load. The baseline pupil size prior to the pupil response (e.g., Aston-Jones and Cohen, 2005) and the peak pupil latency (e.g., Hyönä et al., 1995) provided us with more information about how and when cognitive resources were deployed. We hypothesized that the pupil dilation response would be larger when listening to two sentences compared to one because of additional cognitive load related to the recruitment of more attentional processes. In line with the load theory (Lavie et al., 2004), high cognitive load should result in a drop in performance.

## 2. Methods

### 2.1. Participants

Twelve young-adults (age between 21 and 26 years, mean age 26.0 years) recruited at the VU University medical center where included. To ensure normal hearing, we measured pure tone hearing thresholds at the frequencies 250, 500, 1000, 2000, and 4000 Hz for each participant prior to the experiment. All subjects had normal hearing, defined as thresholds less than or equal to 20 dB HL at these frequencies for both ears. All participants reported normal or corrected-to-normal vision, had no history of neurological disease, were native Dutch speakers, and provided written-informed consent in accordance with the Ethics Committee of the VU University Medical Center.

### 2.2. Task and materials

Everyday Dutch sentences obtained from an open set (Versfeld et al., 2000) were used in this study. Two different sentences were presented simultaneously, one to each ear. One of the sentences was spoken by a female voice, the other by a male voice. The presentation side of each voice was randomized over trials. Speech levels were fixed at 55 dB SPL and sentences were presented in fluctuating noise or in quiet. Independent samples of fluctuating noise were added to each ear. The fluctuating noise modulations mimicked the intensity fluctuations of speech. The fluctuating noise was created by multiplying a noise signal by the envelope of the speech of a single male voice (Versfeld et al., 2000) for two separate frequency bands below and above 1 kHz (Festen and Plomp, 1990). For each trial, different random samples of fluctuating noise were selected for each ear from a 5-min sound file. The long-term average spectra of the fluctuating noise and the Dutch sentences spoken by the male voice were matched to that of the Dutch sentences spoken by the female voice (for a full description see Versfeld et al., 2000). The signal to noise ratio (SNR) was fixed at one of 4 levels (−9 dB, −3 dB, +3 dB, and quiet) by changing the levels of the fluctuating noise relative to the level of the speech (signal), and by presenting no noise in the quiet level. The onset of the fluctuating noise was 3 s prior to the onset of both sentences and continued for 3 s after the end of the longest of the two sentences.

In the ‘single-sentence’ condition, participants were asked to report the sentence spoken by the female voice and ignore the sentence spoken by the male voice. In the ‘dual-sentence’ condition, participants were instructed to first report the sentence spoken by the female voice (S1) after which they had to report the sentence spoken by the male voice (S2). Note that in both conditions the stimuli were identical; only the instructions to the subjects differed across conditions. Although S1 and S2 had the same onset, they were not matched in length. Therefore, the longest presented sentence determined the length of each trial. For the female-voice sentences, the mean duration was 1.9 s (range = 1.3–2.7 s, SD = .26 s) and for the male-voice sentences, the mean duration was 2.0 s (range = 1.3–2.9 s, SD = .30 s). At the end of each trial a 1000 Hz prompt tone was presented for 1 s, after which participants were allowed to respond. Additionally, there was a control condition in which only one sentence, spoken by the female voice, was presented to one ear. Participants had to report that sentence. Again speech was masked by fluctuating noise maskers presented at both ears at three different SNR’s and in quiet. The presentation side of this sentence was randomized over trials.

A sentence was only scored as correct when all words were repeated in the correct order. In case participants could not report the full sentence they were instructed to repeat as the words they had recognized. The proportion of words correct per sentence (proportion correct) was used as a performance measure. The three conditions were presented in a blocked fashion. For each condition two blocks of 28 trials each were presented, containing 7 sentences per level (−9 dB, −3 dB, +3 dB, and quiet). Levels were randomized within each block. Conditions were divided over two blocks in order to keep the presentation duration of each block under 15 min. These six blocks were presented in an alternating order that was balanced over subjects. Six sets of sentences were used for this experiment; these were balanced between subjects over blocks in a Latin square design to ensure that the order of sentences or combination of sentence and block (condition) did not confound results. Before analysis the data of these blocks were combined, which resulted in a total of 14 measurements for each level in each condition. Prior to the experiment, participants were familiarized to the task by listening and responding to 8 practice trials for each condition (the order of these practice trials was also balanced over subjects). The whole procedure, including measuring pure tone hearing thresholds, practicing, fitting the eye-tracker, and performing the actual experiment (with a 15-min break halfway through the experiment), took approximately 1 h and 45 min. Finally, after each block, participants were asked to indicate how much effort it took to perform the SRT task on a rating scale from 0 (‘no effort’) to 10 (‘very effortful’).

### 2.3. Apparatus and procedure

During the experiment, participants were seated in a sound-treated room at approximately 3.5-m viewing distance from a white wall. During the task they had to fixate their gaze to a dot (diameter .47°) that was located at a height of 125 cm on the horizontal middle of the wall. An overhead light source illuminating the wall was placed at 3.5-m distance from the wall outside the participants’ field of view. Light intensity was adjusted so that for each participant the pupil diameter was around the middle of its dilation range. This range was measured by examination of the pupil size in minimum light intensity of 0 lx and a maximum light intensity of about 250 lx. During the SRT task, the pupil diameter of the left eye was measured by an infrared eye-tracker (SMI, 2D Video-Oculography, version 4) with a spatial resolution of .0307 mm and at a 50 Hz sampling rate. Audio in the form of separate files (44.1 Hz, 16 bit) for target sentences and maskers were presented binaurally from a PC by an external soundcard (Creative SoundBlaster, 24 bit) through headphones (Sennheisser, HD 280, 64 Ω) by a dedicated program (written in MATLAB).

### 2.4. Pupil data

For each participant, the mean and SD of the pupil diameter was calculated for each individual pupil trace over a time period starting 1 s before sentence onset and ending at the start of the response prompt for the shortest sentence. Diameter values more than 3 SDs smaller than the mean diameter together with zero values were coded as blinks. Traces for which more than 15% of their duration consisted of blinks were excluded. For the remaining traces, blinks were removed by a linear interpolation that started four samples before and ended eight samples after the blinks. The x- and y-coordinate traces of the pupil center (reflecting eye-movements) were also “deblinked” by application of the same procedure. A five-point moving average smoothing filter was passed over the deblinked traces to remove any high frequency artifacts. A spike detection algorithm was used to detect eye movements on both the *x*- and *y*-traces. This algorithm uses a 100 ms time window sliding with 20 ms steps in which the maximum amplitude differences are calculated between all possible time point combinations within the window. For each participant, the SD was calculated each individual x- and y-trace between the start of the baseline and the response prompt. All trials for which the x- or y-amplitude ranges exceeded 2 SDs were excluded from analysis. All remaining traces were baseline corrected by subtracting a baseline value from each time point within that trace. This baseline value was the mean pupil size within the 1 s period prior to the onset of the sentence (when listening to noise alone or quiet), shown by the left and middle dotted vertical line in Fig. 2B. Average traces were calculated separately for each level within each condition. Within the average trace, mean pupil dilation was defined as the average pupil dilation relative to baseline within a time window ranging from the start of the sentence to the start of the response prompt, shown by the middle and right dotted vertical line in Fig. 2B. Within this same time window, the peak pupil dilation was defined as the largest value relative to the baseline. The latency of the peak pupil dilation (ms) was defined relative to the sentence onset.

## 3. Results

### 3.1. Performance data

**Fig. 1:**
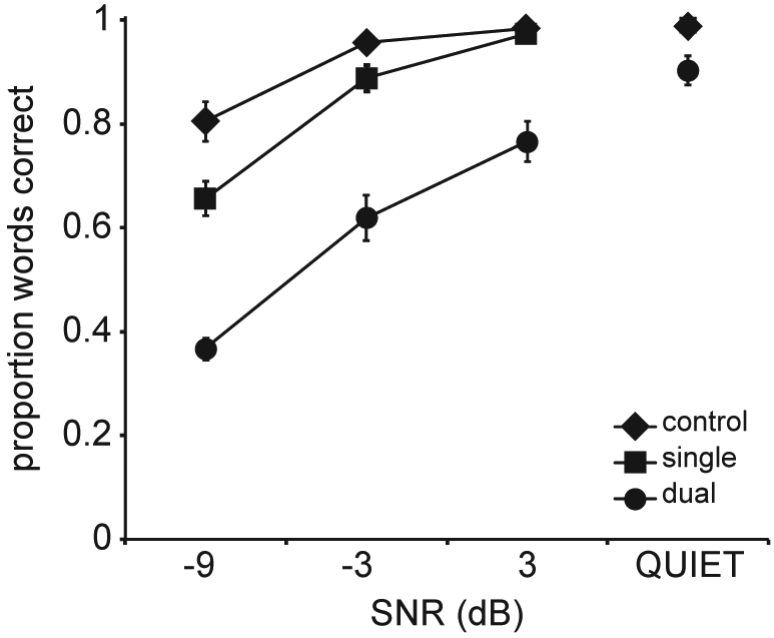
Performance as a function of signal-to-noise ratio (SNR) for each condition, averaged over participants. Error bars indicate the standard error of the mean.

For a direct comparison between performance and pupil response in the dual-sentence condition, we computed the number of correctly reported words across both sentences, divided by the total number of words presented in these sentences. The average performance for each SNR and condition is plotted in Fig. 1. Additionally, the proportion of words correct is presented separately for S1 and S2 in Table 1. For the control condition, performance ranged from .81 (proportion correct) for −9 dB SNR to .98 for +3 dB SNR. For the control condition in quiet, performance was .99. For the single-sentence condition performance ranged from .66 for −9 dB SNR to .97 for +3 dB SNR. In quiet, performance was .99. On average, performance dropped by a proportion of .06 when an additional sentence was presented that did not had to be reported; however, the cost of adding a distracting sentence decreased as SNR increased. At −9 dB SNR performance dropped by a proportion of .15, but in quiet, there was no drop. For the dual-sentence condition, performance ranged from .37 for −9 dB SNR to .77 for +3 dB SNR, and reached .90 in quiet. On average, having to report both sentences, rather than ignoring the male voice sentence, caused performance to drop by a proportion of .21.

A two-way ANOVA on performance (excluding speech-in-quiet) showed a main effect of condition (F_[2,22]_ = 177.95, *p* < .001), a main effect of SNR (F_[2,22]_ = 136.53, *p* < .001), and an interaction between condition and SNR (F_[4,44]_ = 8.341, *p* < .001). Performance decreases significantly both when two sentences need to be processed instead of one and when SNR becomes more negative. The interaction seems to relate to a ceiling effect in the control and single-sentence conditions at the highest SNR level and in quiet. Post-hoc Bonferroni-corrected paired-samples *t*-tests showed that all conditions differed significantly (*p* < .001) from one another and that performance at all SNRs differed significantly (*p* < .001) from one another. A one-way ANOVA performed separately on the average scores for the speech-in-quiet trials showed a main effect of condition (F_[2,22]_ = 10.273, *p* < .01), indicating that the observed condition effect is present even when the speech is not masked. Post-hoc analysis revealed significantly lower performance for the dual-sentence condition compared to the single-sentence (*p* < .01) and control conditions (*p* = .013), and no significant difference between the single-sentence and control condition.

### 3.2. Pupil data

**Fig. 2:**
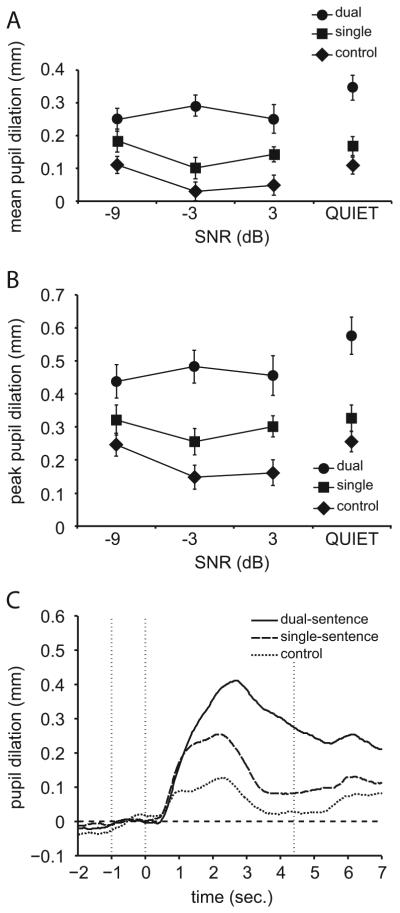
(A) Mean pupil dilation and (B) peak pupil dilation as a function of signal-to-noise ratio (SNR) for each condition, averaged over participants. Error bars indicate the standard error of the mean. (C) Pupil responses per condition averaged over SNR and participants. The onset of the sentences is at 0 s. The baseline, calculated as the average pupil diameter over 1 s preceding the start of the sentence, is shown by the dashed horizontal line. The time window over which the mean pupil dilation was computed corresponds to the range between the second and third dotted vertical lines.

Unreliable pupil traces as a result of a large number of eye blinks (on average 2.9% of the traces) and/or large eye movements (on average 6.8% of the traces) were removed from further analysis. For the remaining traces, the across-subject average mean and peak pupil dilation, calculated separately for each condition and SNR, is plotted in Fig. 2A and B. Average mean dilation, peak dilation, peak latency, and baseline values are presented in Table 1. The average pupil traces for each condition, averaged first overall SNRs (except in quiet) and then across subjects, are plotted in Fig. 2C.

A two-way ANOVA on the mean pupil dilation, with the speech-in-quiet trials excluded, showed a significant effect of condition (F_[2,22]_ = 38.52, *p* < .001), an effect of SNR (F_[2,22]_ = 5.36, *p* = .013), and no significant interaction between condition and SNR (*p* = .082). This indicates that mean pupil dilation, a physiological marker of the mean cognitive load, increases significantly when two sentences need to be processed instead of one, and when SNR becomes more negative. A one-way ANOVA performed separately on the mean pupil dilation values for the speech-in-quiet trials showed a main effect of task (F_[2,22]_ = 18.91, *p* < .001), indicating that this effect also occurs when the speech is not masked. Post-hoc Bonferroni-corrected paired-samples *t*-tests showed significant differences between all conditions in quiet with the exception of control vs. single-sentence conditions (*p* = .191).

Analysis by means of a two-way ANOVA on the peak pupil dilation data, with the speech-in-quiet trials excluded, showed a significant effect of condition (F_[2,22]_ = 48.14, *p* < .001), no effect of SNR (*p* = .057), and no significant interaction between condition and SNR (*p* = .117). This indicates that peak pupil dilation, a physiological marker of the maximum cognitive load, increases significantly when two sentences need to be processed instead of one, but is not affected by SNR. Additionally, a one-way ANOVA performed separately on the peak pupil dilation values for the speech-in-quiet trials showed a main effect of task (F_[2,22]_ = 24.18, *p* < .001), indicating that this effect also occurs when the speech is not masked. Post-hoc analysis (Bonferroni-corrected) showed significant differences between all conditions in quiet with the exceptions of control vs. single-sentence conditions (*p* = .182).

To get more information about how and when cognitive resources were deployed, peak latency and pupil baseline data were analyzed. A two-way ANOVA on peak latency showed a significant effect of condition (F_[2,22]_ = 12.92, *p* < .001), no effect of SNR (*p* = .076), and no significant interaction between condition and SNR (*p* = .159). Post-hoc Bonferroni-corrected paired-samples *t*-tests showed significantly higher peak latency values for the dual-sentence condition compared to the single-sentence (*p* = .01) and control conditions (*p* = .01) and no significant difference between the single-sentence and control condition. This indicates that the dual-sentence condition required a longer processing time than the control and single-sentence conditions. A one-way ANOVA performed separately on the peak latency values for the speech-in-quiet trials showed no effect of condition (*p* = .167).

Finally, a two-way ANOVA on the pupil baseline showed a significant effect of condition (F_[2,22]_ = 7.75, *p* < .01), on SNR (F_[2,22]_ = 5.04, *p* = .016), and no significant interaction between condition and SNR (*p* = .415). Post-hoc analysis (Bonferroni-corrected) showed a significant difference in the baseline measures between the control and dual-sentence conditions when averaged over SNR (*p* = .01), and between the single-sentence and dual-sentence conditions (*p* < .01), but not for any other condition pairs. However, post-hoc analysis (Bonferroni-corrected) between SNR levels (averaged over conditions) showed no significant effects. A one-way ANOVA performed separately on the pupil baseline values for the speech-in-quiet trials showed no effect of condition (*p* = .087).

### 3.3. Subjective data

A one-way ANOVA performed on the subjective effort scores (see Table 1) showed a main effect of condition (F_[2,22]_ = 61.72, *p* < .001). Although the speech-in-quiet trials were also included in the participant’s judgment, the results shows that subjective effort was lowest during the control condition and highest during the dual-sentence condition, in line with the observed pupil responses.

## 4. Discussion

The aim of this study was to investigate how divided attention affects the pupil response to speech processing. In the dual-sentence condition, where two sentences had to be processed, the mean and peak pupil dilation were significantly larger than in the single-sentence condition, where only one sentence had to be processed. Mean pupil dilation was affected by SNR, which is in line with previously reported effects of SNR (when performance was fixed) on the pupil response (Zekveld et al., 2010) and might reflect changes in working memory load (Lunner and Sundewall-Thorén, 2007; Rönnberg et al., 2013). To our knowledge, this study is the first in which the pupil response provides us with a strong indication that dividing attention over two sentences instead of focusing on one sentence results in increased cognitive load when listeners must process speech in noise. According to the load theory (Lavie et al., 2004), high cognitive load arising from increased task demands should lead to a drop in performance, an effect we observed in the current results. However, load theory predicts that selective attention is affected by cognitive load related to additional task demands. Based on the current results we would like to extend this theory, hypothesizing that dividing attention leads to increased cognitive load. Note that in the current experiment, we found no evidence for interactions between how cognition is affected by changes in working memory load, as manipulated by changing the SNR, and the effect of divided attention.

The current behavioral results are similar to those of Best et al. (2010). Performance dropped as task complexity increased (from control to single-task to dual-task) and as SNR decreased. The overall drop in performance in the dual-sentence condition is partly explained by a drop in performance on S1, but also by a relatively low performance on S2 in comparison to S1, as shown in Table 1. The observed differences in S1 and S2 performance in the dual-sentence condition were explained by Best et al. (2010) in two ways. Both explanations assume that information is processed serially (Broadbent, 1958). The first idea is that S2 needs to be stored in echoic memory while S1 is processed to extract meaning. This storage is assumed to be volatile and to degrade with time, causing information to get lost as S1 is processed, and performance to drop. The other explanation suggests that because the response for S2 is always generated after the response to S1; the content of S2 must be stored in working memory for a longer time than the content of S1. Here, we find that the total amount of information successfully processed and reported (as measured by the total sentences correct score) is statistically the same in the single- and dual-sentence conditions when speech is masked by noise. However, cognitive load, as reflected by the pupil response, increases substantially during divided attention as compared to selective attention in order to maintain this same level of total performance. This serial processing as suggested by Broadbent could explain why peak latency was longer for the dual-sentence condition compared to the single-sentence condition. The current results suggest a delay during storage rather than retrieval, as differences in the pupil peak latency were observed while participants were listening to the sentences. Previous research also shows pupil dilation latency to be affected by language, task difficulty, and type of task (e.g., Hyönä et al., 1995; Kramer et al., 2013).

The increase of masker level resulted in a drop in performance, which is in line with previous studies on the effect of masking (e.g., Festen and Plomp, 1990). Additionally, a higher masker level resulted in larger mean pupil dilation. These results seem to contradict the load theory, which states that high perceptual load should result in better performance. As mentioned earlier, increased perceptual load does not necessarily affect target processing the same way in the auditory and visual domains. The hypothesis that high perceptual load results in better performance was confirmed by Lavie et al. (2004) by using a design in which they manipulated the number of visual non-target related items in a visual discrimination task. Note that these manipulations of perceptual load might not directly translate to the manipulations of SNR or by adding a distracting sentence as in the design by Best et al. (2010). While visual perceptual load can be increased by adding elements to the visual scene that can be processed in parallel, adding auditory elements to the auditory scene often results in masking because of spectral overlap, which complicates parallel processing. This energetic masking results in deterioration of the signal, which affects working memory load (Lunner and Sundewall-Thorén, 2007; Rönnberg et al., 2013). A drop in performance for lower SNR conditions in the results of Best et al. (2010) and in the current results suggests that masking could have had an influence on cognitive load. Note that during both the single and dual-sentence conditions, perceptual load based on both the number of presented sentences and SNR was, on average, the same. Hence, the effect of dual-task on performance could still be independently observed.

Remarkably, the pupil response was not smaller for speech in quiet than for speech in noise. On the contrary, for the dual-sentence condition, the largest pupil response was observed in quiet. When there is no interfering sound participants seem to be better able to process both sentences. Also, in the control condition the pupil response was not smaller for speech in quiet than for speech in noise. In contrast to previous studies where speech was presented from a fixed location (Kramer et al., 1997; Zekveld et al., 2010), in the current study the location of the upcoming speech signal was uncertain; this uncertainty may have increased cognitive load. However, this does not directly explain the observed difference in pupil response for speech in quiet compared to speech in noise, because in both cases location was random. Additionally, speech-in-quiet trials were mixed together with speech-in-noise trials that formed the majority of all trials. The peak pupil dilation in quiet might have been affected by the fact that participants anticipated the onset of the masker sound, while instead the target onset occurred. In the latter case, there was no masker onset that could have been used as a temporal cue for the target onset.

We observed an effect of condition across SNR’s on the pupil baseline, which also indicates that *anticipated* task difficulty affects how resources are allocated in a block. According to Aston-Jones and Cohen (2005), a higher baseline (as observed in the dual-sentence condition) relates to a tonic or “exploratory” mode of processing information. During the tonic mode, the firing rate of the locus coeruleus is high, which makes the cognitive system more sensitive for general input. In such a state, we are open to receive other types of stimuli and do not feel that the rewards of focusing on one task are worthwhile, which can result in task switching. A lower baseline (as in the single-sentence condition) relates to a *phasic* or “focused” mode (Aston-Jones and Cohen, 2005). Lower firing rates in the locus coeruleus result in higher sensitivity to more carefully filtered and selected information. In general, a higher baseline relates to a more alert system; for instance, differences in baseline between individuals correlates negatively with fluid intelligence (Meer et al., 2010).

To conclude, the current results suggest that attentional processes strongly contribute to cognitive load during speech processing in complex listening situations. However, although the results show that divided attention by means of task instruction affects the pupil response, we cannot fully exclude changes in the demand of resources other than attentional resources. For one, we know that the pupil response is sensitive to working memory demands, which were manipulated by SNR. When divided attention results in the processing of more information, which was the case for speech in quiet, working memory demands will inevitably be higher. Still, the magnitude of the pupil response in the dual sentence conditions was considerably larger than the pupil response observed in our previous studies for sentences presented with different masker types (e.g., Koelewijn et al., 2012a) or words presented in noise (Kramer et al., 2013). Hence, the current findings seem to indicate that dividing attention over two incoming streams of speech increases cognitive load compared to attending to just one. More attentional resources, and possibly working memory resources, need to be allocated in order to process all incoming information. However, in adverse listening situations, this will still lead to a drop in performance.

## Figures and Tables

**Table 1:** The average performance scores, mean dilation values, peak dilation values, peak latency values, and subjective effort scores as a function of SNR (with the exception of subjective effort) scores and Quiet for each condition.

	SNR			Quiet
	−9	−3	3	
Performance	Proportion words correct (SD)			
Control	.81 (.14)	.96 (.04)	.98 (.03)	.99 (.03)
Single	.66 (.11)	.89 (.09)	.97 (.03)	.99 (.01)
Dual	.37 (.07)	.62 (.15)	.77 (.13)	.90 (.10)
Dual-S1	.61 (.12)	.79 (.15)	.89 (.07)	.95 (.05)
Dual-S2	.13 (.07)	.45 (.19)	.64 (.21)	.86 (.16)
Pupil	Mean dilation (SD), mm			
Control	.11 (.09)	.03 (.10)	.05 (.11)	.11 (.09)
Single	.19 (.12)	.10 (.11)	.14 (.08)	.17 (.10)
Dual	.25 (.12)	.29 (.11)	.25 (.15)	.35 (.13)
	Peak dilation (SD), mm			
Control	.25 (.12)	.15 (.13)	.16 (.14)	.26 (.11)
Single	.32 (.16)	.26 (.14)	.30 (.11)	.33 (.14)
Dual	.44 (.17)	.48 (.17)	.46 (.21)	.58 (.20)
	Peak latency (SD), sec.			
Control	2.55 (1.08)	1.62 (.86)	1.96 (1.14)	3.15 (1.47)
Single	2.30 (.84)	1.96 (.54)	2.01 (.56)	2.41 (1.14)
Dual	3.02 (.90)	2.92 (.67)	2.54 (.45)	3.14 (.71)
	Baseline (SD), mm.			
Control	4.76 (.64)	4.71 (.68)	4.67 (.61)	4.65 (.57)
Single	4.76 (.74)	4.72 (.77)	4.72 (.71)	4.76 (.77)
Dual	5.05 (.72)	4.90 (.68)	4.92 (.71)	4.90 (.72)
Subjective effort	Scores (SD) (low = 0 – high = 10)			
Control	3.12 (1.68)			
Single	4.98 (1.45)			
Dual	7.77 (1.40)			
